# “It’s Still an Animal that Died for Me.” Responsibility and Meat Consumption

**DOI:** 10.5964/ejop.v15i4.1854

**Published:** 2019-12-19

**Authors:** Fabienne Gfeller

**Affiliations:** a Institute of Psychology and Education, University of Neuchâtel, Neuchâtel, Switzerland; Webster University Geneva, Geneva, Switzerland

**Keywords:** responsibility, positioning, vegetarianism, meat consumption

## Abstract

The aim of this paper is to explore the way people engaging in a more or less strict reduction of their consumption of food of animal origin (de)construct their responsibility regarding the food production and distribution system. Starting from a description of the crisis in meat production, it contributes to the understanding of the way people who are sensitive to these issues position themselves by focusing on the notion of responsibility. Ciarán Benson’s work on positioning serves as theoretical background. Through the analysis of interviews and a qualitative experiment with people who changed their consumption of food of animal origin recently, several dimensions along which responsibility is constructed are identified. Those are 1) who bears responsibility, 2) towards whom or what, 3) the action that is considered, 4) the knowledge implicated and 5) the power to act in that situation. The main proposition of the paper is to enhance Benson’s approach through the inclusion of a collective “we.” The study took place in Switzerland, where meat consumption is the norm. This context also implies a certain room for maneuver in the choice of products, as well as the presence of debates around the ecological and ethical implications of meat production.

How do we navigate the complex worlds we live in and take position towards complicated and sometimes unclear issues? More particularly, how do we construct and negotiate responsibilities in relation to these issues? Benson’s work (2001) provides an interesting theoretical approach to these questions, and my proposition in this paper is to draw on it in order to analyze positioning dynamics towards meat consumption. While Benson is particularly interested in rather exceptional experiences such as torture and concentration camps on one side, and artistic experience on the other side, I will use his approach to consider more daily activities, namely those related to eating (choosing products, buying, and so on). Currently, meat consumption is undergoing a crisis. This notion comes from the Greek *krisis,* which can be translated by “judgement, ripping, choice, decision” (Ernst-Vintila, Delouvée, & Rouquette, 2010, p. 517, my translation). Nowadays, the crisis can be understood

in its objective aspects [as] a rupture of balance, a crucial state where a decisive change is imminent, whose outcome can clearly be highly undesirable. It can take the form of an event, eventually social or with social consequences, characterized by contradictions or uncertainty^1^ (Ernst-Vintila et al., 2010, p. 518, my translation).

In addition to that, and in order to have a complete understanding of the crisis, it is essential to also take in account its subjective dimensions, namely how people think about it (as in Ernst-Vintila et al., 2010). In the next section, I clarify why I use this notion in the case of meat production by presenting the objective dimensions of the crisis. Then, I propose a short review of the ways psychologists addressed these issues. After what, my contribution focuses on the subjective dimensions. For this purpose, I draw on Benson’s work on positioning and responsibility. After summarizing Benson’s theory, I present some data and results from a research project on change in foodways, and discuss their meaning in relation to the theorization of responsibility. The aim of this paper is twofold. Firstly, it contributes to the theorization of positioning, in particular through the deepening of the understanding of responsibility. Secondly, it provides preliminary empirical evidence that supports the theoretical discussion. I propose 1) that Benson’s approach is a relevant theoretical tool in order to address psychological activity in times of crisis and 2) that the understanding of responsibility can be enhanced by examining the dimensions along which it is constructed.

## Crisis in the Meat Production System

Three main components participate to the crisis in meat consumption. Firstly, agriculture, and particularly livestock farming play an important role in climate change (McIntyre, Herren, Wakhungu, & Watson, 2009; Wynes & Nicholas, 2017). The problem does not lie only in the carbon emission by the cattle, but also in the transport of animals and in the production and transport of fodder (Steinfeld et al., 2006). Meat production also raises environmental issues less directly linked with climate change, and the Food and Agriculture Organization points to the “very substantial contribution of animal agriculture to climate change and air pollution, to land, soil and water degradation and to the reduction of biodiversity” (Steinfeld et al., 2006, p. iii). The title of the international assessment initiated by the World Bank and the Food and Agriculture Organization, “Agriculture at a crossroad,” clearly falls into the definition of crisis as a moment of imminent decisive change (“crossroad”). Moreover, the report indicates “highly undesirable outcomes” (to use again Ernst-Vintila et al.’s words) such as hunger, climate change, environmental degradation and social inequalities.

A second debated aspect of meat production are the animals’ living and dying conditions. These issues are at the heart of animal ethics, a field of philosophy that “tries to justify the belonging of animals to the moral sphere and to establish the norms of behaviour that we should respect towards them” (Le Goff, 2012, p. 33, my translation). Although a multiplicity of actors defend different perspectives as for instance the “welfare” position (animal faming is morally acceptable if the conditions are good enough) or the abolitionist position (against any kind of animal exploitation), there is a general agreement around the denunciation of the current industrial food production system (Josse, 2013; Kymlicka & Donaldson, 2011; Manceron, 2012), which is also reflected by the consumers’ preoccupations (Kjaernes & Lavik, 2007). These issues are increasingly present in Western European countries (Kymlicka & Donaldson, 2011; Larue, 2015), and different historical changes played a role in their construction. First of all, at least since the 17th century, farming and slaughtering moved to the margins of society. Everything that reminds of the link between meat, the animal and its death tended historically to be more and more hidden and unacceptable (Kjaernes & Lavik, 2007; Larue, 2015), which contributed to a growing tension between the knowledge that meat implies death and the unacceptance of this death. Secondly, the massive industrialization of farming is linked with a deterioration of the animals’ living conditions (Porcher, 2011). Thirdly, the relations between humans and animals are currently changing, a modification that originates in paleontology, primatology, animal protection and liberation, as well as cinema (Celka, 2009) These three changes echo the “rupture of balance” mentioned in the definition of crisis, while the lack of agreement beyond the general denunciation of industrial production shows the uncertainty of the outcome.

A third dimension of the crisis is the health aspect. In this case, the rupture of balance and the undesirable outcome are very visible when a sanitary crisis such as bovine spongiform encephalopathy breaks out. However, a more general overconsumption of meat (Office fédéral de la sécurité alimentaire et des affaires vétérinaires, 2017) also equates to a rupture of balance and undesirable outcomes. Health questions become a collective issue when it comes to the discussion of hygienic norms and regulations in food production (Levenstein, 2012; Masson, 2011). Moreover,

[although] individuals may see these concerns as distinct, in reality they are closely related—largely stemming from the mass production of animals as food that have characterized meat production in the US and to a lesser extent, around the world, for several decades (Grauerholz & Owens, 2015, p. 568).

This quotation also highlights that these issues are not ahistorical. Animal ethics, as a moral reflection on relations between humans and animals, can be traced back to Antiquity (Larue, 2015). Their entry into the legal sphere is nevertheless a contemporary topic (Kymlicka & Donaldson, 2011). The discussion on the environmental issues is currently particularly vivid in relation to climate change, and the bestseller *Diet for a small planet* (Lappé & Hahn, 1971) is a forerunner of these debates. Nevertheless, this issue has a much longer history, and Belasco (2006, see chapter one) traces it back to Socrates. In other words, the debates are not new, but the huge changes that the agricultural system undergoes since the second part of the 20th century (Porcher, 2011) produce a specific situation that presents its own challenges. In Switzerland for example (where this study took place), the number of farms get down from 79,000 in 1996 to 52,300 in 2016, a decrease of 34%, while the surface dedicated to agriculture increased. The number of pigs stayed stable over this period, while 60% of the farms active in this sector disappeared (Meyre, 2017). These few numbers show the radical changes the sector is undergoing. Farming becomes more and more industrial, while small and middle size farms tend to disappear (McIntyre et al., 2009). This is without mentioning other issues such as working conditions (Droz & Forney, 2007; Garrouste & Mitralias, 2013), conflicts around patents on life (Then, Tippe, Batur, Lapprand, & Meienberg, 2016) or lack of trust in the production system (Larue, 2015; Levenstein, 2012).

## Contributions from Psychology

Eating is not optional (or not for long time) for human beings and we are dependent on the state of our planet in order to live—these are just biological conditions to which we cannot escape, although we can modify the shape these conditions take, and our possibilities in it. Without denying that the food production system is technically, biologically and chemically complex and needs to be studied by researchers in these areas, it is also a matter of human activity and to that extent, social sciences are indispensable to face the challenges in this area (Forney, 2013; Porcher, 2011; Ziegler, 2011). How does psychology contribute to this area?

The study of the relation to environment was present in psychology from the beginning, but what was meant by this notion evolved depending on the main paradigms and the dominant social concerns (Morval, 2007). Starting during the 60’s, green environmental psychology is a reaction to issues such as “conservation of nature, of energy, of resources, pollution, industrial and technological risks, ecological ‘responsible’ behavior and everything that has been called a ‘green’ movement” (Pol, 1993, p. 165). Nowadays, there is a focus on attitudes and behaviours (Fielding, Hornsey, & Swim, 2014; Swim et al., 2011), and many studies aim to establish correlations between some “values” and a pro-environmental behaviour (see for instance Gatersleben, Murtagh, & Abrahamse, 2014). There is nevertheless a lack of understanding of how these values and attitudes are constructed and navigated. Many studies presuppose a clear distinction and a simple causal link between values/attitudes and actions/behaviour. These presuppositions might be criticized as reductive conceptualizations (see Branco & Valsiner, 2012). It seems that more systemic considerations of the relations between representations, values or consciousness and the current ecological crisis rather take place in other domains than psychology (see for instance Bourg, Roch, & Bastaire, 2010; Latour, Schwartz, & Charvolin, 1991; Orr, 1994).

Psychologists who studied ethics, values or morality usually focus on humans (see for instance Branco & Valsiner, 2012; Gibbs, 2014; Kohlberg, 1981). Relations to animals are rather rare as an object of study, although this topic seems to emerge recently. Kasperbauer (2017) provides an overview from which he concludes the existence of a link between attribution of phenomenal experience and moral consideration, while the correlation between attribution of agency and moral consideration is rejected.^2^ Relations between humans and animals have also been studied under the angle of disgust, and researchers highlight the central place of animals in what provokes disgust. A first interpretation suggests that disgust towards animals has the function of protecting us from diseases; a second one is that humans are disgusted by anything that reminds them of their animal condition and therefore of their mortality (Kasperbauer, 2015; Rozin, Haidt, & McCauley, 2008). Nevertheless, this would explain avoiding meat rather than its consumption, especially in contexts where it is not essential for survival. Moreover, although raising animals and eating their meat are certainly deeply enrooted in primal survival mechanisms, these are also highly complex cultural and social activities that cannot be reduced to survival issues (Anderson, 2005). Therefore it becomes important to study the meanings involved in these relational activities (Porcher, 2011), which, as any relation, imply ethical dimensions (Marková, 2003).

In this paper, I explore meat consumption and vegetarianism through the theoretical perspective of positioning dynamics. Although the first part of this text largely highlighted the problematic aspects of meat production, the situation is not completely black and white and the arguments raised above do not clearly imply a strict vegetarian diet (see also Bruckner, 2016). Environmental and ethical reasons might as well lead to a reduction of meat consumption, or to the exclusion only of certain types of meat, for example stopping to eat beef, considered as the most environmental harmful meat. Some even use the environmental argument against vegetarianism, claiming that in permaculture animals can be an important part of a global system. On the ethical aspect, if it is the treatment of animals in industrial farming that is considered as problematic, then eating animals raised in proper conditions might be acceptable or even encouraged (Porcher, 2016). Moreover, there are plenty of other aspects that enter into people’s decisions, such as the wish not to offend a host who prepared some ragout, the pleasure of eating a steak, the availability of meatless products or the reactions of family members. In other words, although the current meat production system is clearly problematic and even dangerous, the action to take with regards to this situation is not evident, and people who face these issues need to navigate a complex landscape. Dafermos, Triliva, and Varvantakis (2017) highlight the risk that situations of crisis hinder or distort psychological development. However, through the use of cultural signs allowing to understand and transform the situation, individuals can also become “masters of their own behavior” (for which they borrow Vygotsky’s words, see Vygotsky, 1978b). In a similar way, my hope is to contribute to a better understanding of possibilities of complex psychological processes in crisis situation, and more precisely to what extent people can become reflective actors in that particular situation. As I argued elsewhere, this situation calls for creativity (Gfeller, 2019) which can be conceptualize as a (re)positioning. I will not expand on the creative dimension here, but I will continue on the notion of repositioning, with a focus on responsibility. In order to do so, I will draw on Benson’s work, which will be the object of the next section. It is however beyond the scope of this article to provide a review of different ways to conceptualize positioning, to discuss further the choice of this notion in relation to alternative ones or the focus on Benson in particular. The range of this article is more modest and consists into the proposition of one theoretically grounded way to address the above-mentioned issues.

## Positioning: Navigation and Responsibility

According to Benson (2001), one of the main functions of human mind is to navigate the worlds we live in. “A fundamental problem confronting every one of us, and indeed every sentient creature, is how to position ourselves in the worlds we inhabit and how to find our way around them” (Benson, 2001, p. 3). In that sense, the self is conceptualised as a locative system allowing humans to orient themselves in complex landscapes and to actively navigate them. This includes both a physical level, in which we perceive and act on the material world, and a semiotic and symbolical level in which we dialogue with multiple discourses, we make the world and our experience meaningful and construct continuity in our lives through narrative activities. Both levels are always present and are tidily interrelated. For instance, the discourses we encounter are conveyed through physical means, be it written signs or sound waves, and psychological processes could not unfold without the brain (see Benson, 2001, chap. 2; Toomela, 2009). This conceptualization of human self as a locative system in both physical and symbolic worlds makes Benson’s approach relevant to address such a complex issue as food choices and habits. Indeed, these activities are at the same time strongly embodied (eating is—together with breathing—one of the human activities in which the physical exchange between the outer material world and the human body is the most salient) and highly cultural (Anderson, 2005), social (Fischler, 2013) and symbolic (Douglas, 1966).

The possibility to navigate and to position oneself in a landscape is tidily related to the notions of choice and of responsibility, which are constitutive of self-creation, the possibility to create one’s own path in the landscape. “One system of belief that enables the idea of self-creation is that which includes central beliefs about personal responsibility and personal powers to choose one’s own moral and intellectual path, subject of course to certain constrains” (Benson, 2001, p. 122). Every person develops a sense of him or herself as being a certain kind of person, which entails both ideas about what one possesses, but also of what one can or cannot do. This does not only involve the technical possibility, but concerns also the idea of moral boundaries. There are some actions that are so fundamentally in conflict with one’s self-meaning, with this idea of the kind of person I think I am, that they are unthinkable. There evocation is strongly linked with such emotions as disgust, shame/contempt and anger/anxiety, depending on the type of moral system we rely on (Benson, 2003; Shweder, Much, Mahapatra, & Park, 1997). These emotions function as attractors and as “moral compass” (Benson, 2001, p. 131) in the navigation process. On the semiotic, discursive level, morality is omnipresent. For Benson (2001), “morality is an inescapable condition of selfhood, […] just as it is for peaceful, tolerant, pluralistic society” (p. 126). Children learn about morality as they learn from their community what matters or not, what it is worth to care about or not.

Responsibility can be distinguished between other-responsibility and self-responsibility, the later being defined by Benson as “acknowledging and holding my-self accountable for the consequences of actions initiated by myself” (Benson, 2001, p. 132). Both are negotiated in interactions in which duties and rights are attributed. Moreover,

our disposition to assume responsibility for our actions and their consequences, to be self-responsible, develops from our own positioning as children. This in turn depends upon how our community constructs its version of ‘childhood’ and therefore on its own particular ‘make-a-person’ practices (Benson, 2001, p. 136).

Responsibility is attributed in relation with agency, in other words it is related with a certain power over the situation, with possibilities to act in another way (see also Driver, 2016). This raises quite complicated issues of relations between actual and perceived possibilities that imply also a temporal dimension. Benson (2001, see chapter 10) provides extensive examples from cases of extreme suffering. He shows that victims often consider themselves as responsible for what happened and think they should have acted otherwise or should have known, although this was obviously not possible at the time they refer to, while oppressors consider that they cannot be hold for responsible for their actions.

In relation to food consumption, Austgulen (2014) highlights that individual responsibility is considered as central when it comes to the environmental impact of meat consumption and underlines the neoliberal assumption behind this conception of the consumer, implying that the state does not play a regulating role. However, in the consumer—industry relation, a single individual cannot make any difference through just buying meat or not (Driver, 2016). In that sense, individual responsibility needs to be conceptualized in a way that does not isolate the individual from a larger group, or this is at least what the work of a political scientist (Austgulen) and a philosopher (Driver) imply. In order to deepen the understanding of responsibility in meat consumption from a psychological point of view, I will now turn to the empirical part of this paper.

## Method

The data presented here was collected during a research project on changes in foodways around products of animal origin. Participants are 10 adults who changed their habits of consumption during the previous 2 years (except one participant who changed from a meatless diet towards a diet including meat approximately 5 years ago, but who was included as a contrasting case). This selection is based on the hypothesis that positioning dynamics become particularly visible in periods of transitions, as these involve a transformation of identity (Perret-Clermont & Zittoun, 2002; Zittoun, 2009). Most of the participants reduced their meat, fish and/or dairy products consumption in a radical way, either stopping consuming completely some of these products, or considering their consumption as an exception to their normal diet. Participants are aged between 20 and 70, and their change in food habits relied on different motivations, mainly health, environmental and ethical reasons.

**Figure 1:**
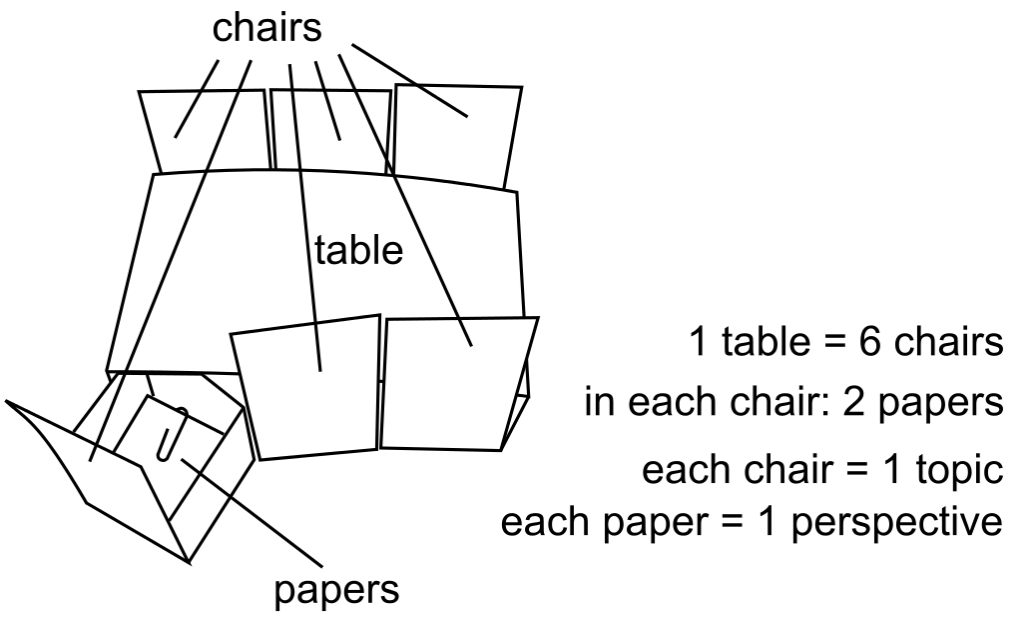
Cartoon table for qualitative experiment.

All participants took part in a narrative interview and in a qualitative experiment. The interview focused on the reasons and unfolding of the change. I followed the structure of a classic narrative interview (Schütze, 1983), nevertheless I asked participants to focus on their foodway and especially on the consumption of products of animal origin. Another point on which I distance myself from Schütze is that I consider the interview situation as co-constructed and the narration as addressed to me and sometimes to a larger audience (see Jovchelovitch & Bauer, 2000). The qualitative experiment aimed at provoking positioning “on the spot,” with a more microgenetic focus. It is inspired by de Saint-Laurent (2018) who used a similar method in order to study historical representations. It has the advantage of prompting positioning on issues that can be emotional in a relatively safe and playful frame. This frame is important as a certain feeling of safety is necessary for people to engage in thinking processes in which they also partially risk their identity (Perret-Clermont, 2004), and the identity certainly is put at stake when the positioning is changing or uncertain. Therefore, this frame was set up in order to increase the chances that these kinds of movement would emerges. Concretely, a small table in cartoon was presented to the participants (see Figure 1). In the chairs, they would find papers with different perspectives on the topic (see Appendix C for the content of these papers), and they were asked to imagine and say aloud what they would answer to someone stating this during a dinner party and what they think about it. The content of the papers was elaborated by myself, drawing on different debates I identified previously. Their aim was to bring in the experimental setting a multiplicity of voices, against which the participant can position her or himself. Both the interview and the qualitative experiment were audiotaped and transcribed (see Appendix B for the transcription conventions).

## Results

Before moving to the analysis per se, I will briefly present the participants’ profiles. One third of the participants contacted me after seeing my call for participants. This means that they give enough importance to that subject to notice the flyer, take the initiative to contact me and be willing to speak about it. This degree of engagement is certainly not representative of everyone’s. The other participants are people who I asked to participate, or who volunteered after discussing with me about my work. Most of them clarified that they are not strict vegetarians, or that they do avoid classifying themselves as vegetarians. Moreover, the participants have very diverse profiles in terms of strictness of habits, type of products of animal origin that are avoided, reasons for change and length of their story in relation to vegetarianism. Nevertheless, all of them are engaged in a form of reflection and concrete change in their food habits, which is not representative of the majority of the population, but which makes them interesting participants. Indeed, in order to study the “how” of the positioning process, there must be a form of navigation of these issues. Although it is not impossible that other people are reflective about these issues and that I could provoke this navigation through the research design, I chose to work with people who have a certain history of engagement with these questions. The study took place in Switzerland, where vegetarian diets are rather exceptional. Depending on the source, they represent 1 to 5% of the population.^3^ This context also implies a certain room for maneuver in the choice of products and the presence of debates around the ecological and ethical implications of meat production.

For the analysis, both parts of the data set (interviews and experiments) were considered as a whole and treated in a similar way, drawing on the assumption that, although elicited through different ways, both parts provide substantial information about movements of positioning related to issues of responsibility. A more differentiated analysis would probably lead to the identification of variations between the kind of positioning in the interview and in the experiment, however this lies beyond the scope of this article. Rather, here the fact to combine both methods is a way to provide more (quantitatively) and diverse (qualitatively) data on positioning. After an initial global reading, the whole data set was coded looking for elements related to responsibility, and in particular for movements of (de)construction of responsibility. This coding led to the identification of several dimensions that play a fundamental role in the construction and negotiation of responsibility. This part of the analysis was conducted drawing on thematic analysis (Braun & Clarke, 2006) with the aim of identifying recurrent patterns in the data set, with an initial focus on responsibility in order to “provide a detailed account of one particular aspect” (Braun & Clarke, 2006, p. 11) of the data set. In addition to this, the selection of a few extracts in which the (de)construction of responsibility is particularly visible allows a more detailed analysis of the articulation of those dimensions in the process of positioning. With this procedure, I come closer to case studies that allow the understanding of dynamic process such as positioning (see Benson, 2010 for an example of analysis). In the following, I present four extracts that were selected because this process of construction is particularly visible and because they illustrate the diverse dimensions identified as relevant during the thematic coding. In the discussion, I elaborate on these dimensions. The original data is in French (except the interview with Lisa, held in English), and the extracts were translated by myself (see Appendix A for the original extracts in French).

### Aurelia: We Cannot Kill Animals Because It Is Tasty

This first extract comes from Aurelia’s reaction to a text in the qualitative experiment stating that the fact that the majority of people does not like the taste of raw meat proves that eating meat is not natural. Aurelia has a strictly vegetarian diet, tends towards veganism and identifies herself as a vegetarian.

**Extract 1: Aurelia (experiment)**

[she just told me that she likes the taste of raw meat]

Interviewer and if somebody tells you that he does not want to be vegetarian because he likes it too much, (.) do you think it’s a good reason?

Aurelia no,: but I know I said it myself so uh: but: I have difficulties to be tolerant with that because I think uh (.) I try to: (.) to make this person understand that it is terrible, we can not: (.) we cannot kill animals because it is tasty, (.) but. it is not even to kill, it is: what we make them endure, uh: (.) in big farming uh it’s mainly that

While she first invalidates the argument she read, after my question, she takes position against the argument that liking meat justifies its consumption by referring to the implications of meat consumption, that is, killing the animal. She further redefines her position by adding that the problem is not that much killing than “what we make them endure” in big exploitations. The condemnation of the argument of pleasure relies on the construction of a relation between the consumption of meat and animals’ suffering. Doing this, she takes position against meat consumption. If according to Benson, self-responsibility means “acknowledging and holding my-self accountable for the consequences of actions initiated by myself” (Benson, 2001, p. 132), Aurelia’s positioning relies on this link between the action initiated by a person (eating meat) and its implications (animal suffering). Should we speak about consequences, as Benson does? We might hypothesize that Aurelia implicitly supposes that eating meat is bad because when buying it, people give money to this industry and this sustains animal suffering and killing. In that sense, the notion of consequence would be appropriate. Nevertheless, nothing indicates such a detailed representation, and the link might very well be conceived in a more general way, such as “eating meat is bad because it logically implies (in the current context) animal suffering that is not acceptable.”

One particularly interesting aspect in this extract is the use of the “we,”^4^ as it might include both Aurelia herself and her virtual interlocutor among the perpetrators who kill and make endure. Moreover, with this use of the “we,” responsibility has a wider scope than strictly personal, nevertheless who exactly is implicated is not further defined. The rest of the data concerning Aurelia makes it clear that she includes herself, positioning herself as responsible and trying to act accordingly. This points towards a first dimension that the thematic analysis allowed to highlight, namely the question of who bears responsibility.

### Lisa: Still an Animal That Has Died for Me

Lisa became vegetarian 5 years ago, and after being strictly vegetarian during a few years, she started to eat meat again occasionally when she got pregnant, and continued after giving birth. She defines herself as a vegetarian but eats meat from time to time. She perceives this as a contradiction and feels unsatisfied.

**Extract 2 : Lisa (interview)**

Lisa I have some some meat (from time to time) because when we buy [her husband and her] or my parents come and bring salami, (2) I- I said ok some is fine,: from time to time, it’s not a big deal, although I: I think it’s not a big deal in terms of you know: the planet, uh and ecological reasons, but uhm ethically speaking I don’t feel good, cause I think (.) ok. still an animal that has died for me,: for just my pleasure to have a small piece of salami it doesn’t change my life this small piece of salami,

In this extract, Lisa establishes links between the consumption of meat, the death of an animal and the environmental impact. She starts by framing meat consumption to specific social situations with her husband and her parents. This is already a way to position herself, saying that although she eats meat, this is limited to a few situations, which implicitly distinguishes her from an average meat eater. She brings in the question of the ecological impact of meat production in order to sustain this position. This dimension is used to diminish personal responsibility, as the main point is that the impact of one piece of salami is negligible. In this way she presents the positioning of being an occasional meat-eater as defendable. In contrast to that, she expresses guiltiness in relation to the death of the animal, which fundamentally questions the previous position. Indeed, the positioning implied here would be that of a strict vegetarian. This extract illustrates well two other dimensions highlighted through the thematic analysis: what is the action that is considered (is buying important or is eating important?) and towards what is the responsibility constructed (here: environment and the killed animal). Guilt can be interpreted here as the emotional reaction to the discrepancy between a position logically considered as good and action. As mentioned by Benson (2001), one important aspect of responsibility is the possibility to act differently, and indeed Lisa had a strict vegetarian diet during a certain time and knows people who still have one.

### Laura: To Raise Awareness Among People

The next extract is related to an already mentioned dimension, namely who bears responsibility. Laura has a long history with vegetarianism, which started during her adolescence when her whole family became pescatarian (avoiding meat but eating fish). She then alternated between pescatarism, strict vegetarianism and veganism. Recently, she started to eat chicken a few times a year.

**Extract 3 : Laura (interview)**

Laura Whereas now, we see it more and more, there starts to be mass products, for vegans, for vegetarians, and then, for me it has the opposite effect, I told myself (.) ok now, (.) because me for instance I love meat. (.) in terms of taste, I love it. It is really an ideological question. I told myself, uh now that I am uh: I am not the only one anymore there is really, this mass of of the new generation that is vegetarian or vegan, I can allow myself to: to make a step: to withdraw a little bit because it’s clear that in the beginning, I was saying I’m vegetarian,: it was also a bit something political, (.) some advertisement, to explain why, to raise awareness among people (.) and that, I don’t feel anymore, the duty to do it. Because I saw that there is a strong new generation there is a strong participation in vegetarianism and veganism.

In this extract, there is a shift from personal responsibility to more shared responsibility, expressed through the use of terms like *the vegans, the vegetarians, the mass of the new generation,* but a collective responsibility in which Laura doesn’t include herself anymore. This extract illustrates how the interplay between individual and collective responsibility can go with a decrease of strictness in personal engagement. Her positioning shifts from a strict personal engagement as vegetarian towards a less clearly delimited position in which she still argues these questions are important, but feels less compelled to act as a strict vegetarian and to promote it. It is indeed the case that issues related to meat consumption are more discussed in the public sphere where she lives nowadays and that many vegetarian options appeared during the last years. Her positioning is implicitly linked to the possibility of change and to an understanding of what are the impacts of different ways to act. In other words, this extract exemplifies the importance of the person’s possibilities of action and how these allow her to affect the world, which constitutes another dimension underlined in the thematic analysis.

### Léa: Well I Don’t Know Vietnamese

The answer to the question of who carries the responsibility might also exclude the person’s responsibility, as in the following extract. While Léa was a person eating meat regularly, 6 months before the interview, she saw a documentary on meat production that deeply shocked her. At the time of the interview, she is still very uncertain about what to do. The following extract is part of a section of the interview in which she speaks about her occasional consumption of meat, and follows a reflection about her eating meat at her parents’ place and the feeling of guiltiness that this implies.

**Extract 4, Léa (interview)**

Léa We went on holidays this summer with my boyfriend, (.) to Vietnam, and they ate a lot of, (.) pork meat. (.) uh there is pork meat everywhere in the well (.) pork but also others but mainly pork and (3) and it is impossible in fact sometimes it is very very hard to::: to say, uh::: well to find something a vegetarian meal, (.) and uh: (.) and also we don’t want to make any trouble:, and uh when we eat in a little uh well often these are family restaurants, where there is only one or two dishes and: and you discover the soup because well I don’t know Vietnamese, I see my soup and I see things looking like meat I won’t start to ask,: uh if I can change and everything then I try to sort but (.) well pork meat then you smell it in the broth, (.) so it is true that (2) I didn’t feel so guilty,

The fact that she expresses feeling no guiltiness (in contrast with situations where she eats meat prepared by her parents) indicates that the feeling of responsibility is not engaged in the same manner than in her everyday context. Interestingly, she insists on the difficulty to find something vegetarian, which reminds us that alternative possibilities are necessary for the emergence of responsibility. Another aspect she mentions is that she does not want to make trouble. This could be interpreted as the interference with another responsibility, that of respect towards people encountered in a different cultural context. In that sense she positions herself as a tourist respectful of the local conditions, a position that prevails over her vegetarian positioning. Moreover, she adds that she does not understand Vietnamese, meaning that the knowledge that would possibly allow her to avoid meat is not available. In short, Léa provides us here with several reasons for which she can not be blamed for her meat consumption in this case, one of them being (lack of) knowledge, which is still another dimension highlighted in the thematic analysis. Who is responsible then? Nothing indicates that the question crossed her mind, but we might hypothesis that the restaurateurs (specific other) or the cultural context (generalized other) could play that role.

## Discussion

The extracts presented above illustrate movements of (de)construction of responsibility. I will now come back on the dimensions that are central to these movements. Firstly, we might ask who bears responsibility, which leads to identify three categories. These are “I,” “we” and “other(s).” Rather than classifying extracts in these categories, it seems more interesting to pay attention to their articulations. Aurelia’s discourse for instance is addressed to a virtual other who does not share her positioning. Her (“I”) responsibility becomes to argue and to convince this other. In order to do this, she refers to a common “we,” including her personal responsibility and the other’s in a shared responsibility. Lisa in contrast focuses solely on her own responsibility, neither her husbands or her parents responsibility (to eat meat themselves or to offer salami to her) are questioned, nor is there any broader we or they (vegetarians, food industry…). Laura articulates the “I” and the “we,” also including her positioning in that of a group. Nevertheless, rather than sustaining her engagement, this inclusion is linked with a reduction of her responsibility. Blaming that movement would nevertheless be too reductive. There are many examples in the data where participants express the feeling of never doing well enough, of being powerless, and one person even refers to psychological troubles linked to the fact that he would always try to take too much on his own shoulders. This brings us back to the issue of the responsibility of the consumer in a complex system where personal responsibility is at the same time highly valued, but means of action at the individual level are small or uncertain, such as underlined by Austgulen (2014) and Driver (2016). In accordance with what Benson (2001) highlights there can be a discrepancy between the felt responsibility, and what would be the field of responsibility based on what lies into the person’s possibilities of action. What emerges from this analysis but does not appear much in Benson’s work is that participants actively try to understand the distribution of responsibilities between themselves as individuals, themselves as members of groups, and others. This distribution appears as fundamental in the construction of responsibility.

A second dimension concerns the entity towards which the responsibility emerges, and this is mainly visible in Lisa’s extract. We saw that both the planet and the animal that died call her to act, nevertheless in slightly different ways. The animal is also an important entity in Aurelia’s example, nevertheless this time it is not that much because he dies, but because of his suffering. In both Aurelia and Laura’s case, we might also say that the other (human being) calls for some action. As she or he is mistaking or ignorant about the implications of meat consumption, there is a need to inform or to convince her or him. Finally, in Léa’s case, we might also suspect that the restaurateur appeals a certain kind of behavior, which is the consumption of what she or he proposes. In this case, this behavior is in contradiction with the one that the suffering animal calls to (Léa became vegetarian for reasons related to animal ethics). Although Benson (2001) does not particularly mention the fact that responsibility is fundamentally addressed, oriented towards something or someone, his understanding of positioning is inherently social. Therefore, this aspect does not contradict Benson’s approach, but constitutes a proposition of deepening of the theorization of responsibility. The fact that participants evoke the environment, animals and humans as entities towards which one is responsible confirms that vegetarianism is an issue anchored in our relations to the planet, to non-human animals and to others (as underlined in the section about crisis), and that it is subjectively perceived as such.

Thirdly, the action that is considered also varies. In Léa’s case, it is unclear if the central problem relies in the act of ordering or of eating the meat. When Lisa specifies that her parents bring salami, this precision seems to act as a reduction of responsibility. In both Laura and Aurelia’s cases, the act to inform seems more important at this point of the interview than what they are buying or eating. Am I first of all a living being eating another (former) living being? A consumer who invests money in certain kinds of networks? A person who can affect other people? Benson (2001) presents the landscape as complex, and so are the social positions and relations that are part of it. Nevertheless, he does not insist much on the possible diversity of positions that a single individual can combine and the potential tensions that these combinations might involve. Informing others as a possible way to enact one’s responsibility leads us to the fourth dimension, namely knowledge. The example of Léa illustrates that the person considers that if she has no access to the relevant information or if simply she did not know, it diminishes the responsibility. Other participants (not presented here) also discuss their responsibility to search for further information, in terms of “one should know” or “I should look for more information.” Lisa also takes the responsibility to inform others, whereas Laura even presents this aspect as one central component of her labelling herself vegetarian. Nevertheless, knowing alone might leave the participants with a feeling of guilt or of disgust. This aspect could be considered as one that falls under the activity of meaning-making (Benson, 2001) which reminds us that it is not simply about getting information, processing and communicating it. More globally, individuals try to make sense of the world they live in and their position in it (Benson, 2001). However, Benson only skims over the responsibility of (not) knowing and the power relations entailed in knowledge. This could be pushed much further (see for instance Gilson, 2011), especially in the case of meat consumption where so many parts of the process are happening far from the consumers’ eyes and other senses. Finally, a fifth dimension are the possibilities of action. This becomes notably clear in Léa’s example, where the fact that they are almost no vegetarian options available makes meat consumption more acceptable to her. Similarly, many participants referred to other places, times or fictional situations in which meat consumption would be acceptable because it is a matter of survival. Nevertheless, it is not only a question of the possibility to act in another way, but also of power to act, which includes the possibility to affect the world through one’s actions. When Lisa says that it is not a big deal for the planet, the central point is that her behavior will not affect the environment in a noteworthy way. Similarly, when Laura speaks about her engagement as political, she points to the possibility to affect other people as an intermediary step to affect the production system more efficiently. This aspect is tidily linked to the relation between responsibility and power over the situation (Benson, 2001). However, while Benson mainly examines this point under the angle of the possibility to act in other ways, in the data examined here, the possible impact of the considered act also plays a fundamental role.

This last example brings us back to the first dimension mentioned in this discussion. This is not a simple coincidence, but is due to the systemic interrelations between all those dimensions. Indeed, the way I understand my own and other’s responsibility as well as what I or we can or should do are tidily linked to the understanding I have of the global situation, the entities that compose it and the way they are related. In Bensons’ (2001) words, we could say that the construction of responsibility can be understood only in relation to the landscape it is part of. Although it is not really the case for the extracts presented above, a few participants presented their positioning by framing it with terms referring to a certain theorization of societal dynamics or ideologies, such as capitalism or antispeciesism. These seem to act as tools allowing a certain understanding of the relations between the entities.

## Conclusion

Starting from the assessment that meat production is currently undergoing a crisis, I presented contributions from psychology in relation to these issues. I proposed Benson’s approach as a relevant theoretical tool allowing to understand how individuals navigate the complex and uncertain landscape that characterizes the crisis around meat, and focused in particular on the notion of responsibility. In the empirical part, I analyze the way responsibility is (de)constructed by more or less strict vegetarians and identified several dimensions along which this (de)construction proceeds, namely 1) who is responsible, 2) towards what/whom, 3) for what action, 4) what do (I) know and 5) what is the power to act in that situation (what can be done and what impact does it have)? I underlined that these dimensions are interdependent, and therefore we might add as a sixth dimension the global understanding of the situation that includes all the other dimensions as well as statements and interrogations about their relations.

As a conclusion, I propose to discuss a few points of Benson’s theory, based on the work presented here. First of all, Benson distinguishes between self and other-responsibility, a pertinent distinction, as the distribution of responsibility between the individual and potential others plays a fundamental role in the person’s positioning towards meat consumption. However, this distinction does not justice to the role of the collective (we) that appears to be central in the data analyzed here. The inclusion in a collective “we” might act as a support for engagement. In such cases, as the example of Lisa illustrates, individual behaviors make sense because they are part of a broader movement. On the other hand, as for instance in Laura’s case, the collective “we” might be related to diminution of individual engagement. This implies that as a researcher one should not only take into account the way the collective is (or not) present, but also how it articulates with the other entities and how the whole system of interrelations is understood.

Secondly, Benson combines semiotic dimensions (acknowledging, holding one-self accountable) with the notions of action and agency. The analysis presented above shows that these two dimensions are indeed central, also when it comes to food choices. In order to navigate the world, the world must be made understandable, and for this it is necessary to have information and to be able to articulate it in a meaningful way. Seeing violent images of slaughterhouses might indeed play a role as a trigger in a repositioning process, as in Léa’s case, and it certainly provides some information about the system, nevertheless this is problematic when it leaves the person with a strong feeling of disgust and/or guiltiness. Disgust in particular often appears in relation to the issues raised by animal ethics, and this raises the issue of the way of communicating about this topic. In order to continue to navigate the food landscape, it seems necessary both to have the tools that allow understanding the interrelations and to envisage possibilities to affect the system. The value of Benson’s conceptualization is also not to fall into a linear understanding, in which first comes the feeling of responsibility, then the more or less appropriate action. On the contrary, Benson includes the issue of possible actions from the beginning, and from our data analysis we suspect a dialectical dynamic between meaning-making processes in mind and action in external world (in vygotskian words, between internal and external processes, see Vygotsky, 1978a). However, this point needs further investigation.

Finally, the notion of consequences of actions used by Benson also warrants some attention. I suggest this notion is perhaps not completely appropriate here as in some cases the problem seems to lie before the action or to be related to it more on the level of logical implication than of real consequence (as for instance when it is considered as ethically problematic to eat a dead animal). One possible explanation would be that sometimes participants reflect in reference to a consequentialist moral approach (in which consequences are indeed central), and sometimes their reflection is nearer to a deontological approach. It seems that Benson’s theorization of responsibility does not include this kind of moral reasoning.

## Appendices

### Appendix A: Original Extracts of Data in French

Extrait 1: Aurélia Intervieweuse ouais. (.) et pis si quelqu’un te: te dit qu’il veut pas être végétarien parce qu’il aime trop ça, (.) tu penses que c’est une bonne raison? Aurélia non,: mais moi je sais qu’j’l’ai eu dit donc euh: mais: j’ai du j’ai du mal à être tolérante avec ça parce que j’me dit euh (.) j’essaie de: (.) de faire comprendre à la personne que c’est terrible, on peut pas: (.) on peut pas tuer des animaux parce que c’est bon, (.) après. C’est même pas tuer, c’est: c’qu’on leur fait subir, euh (.) dans dans dans l’exploitation grand élevage euh surtout ça

(Extract 2 is originally in English)

Extrait 3: Laura Laura Tandis que maintenant, on le voit de plus en plus, on commence à avoir des produits de masse, pour les véganes, pour les végétariens, et là, pour moi ça fait la- l’effet inverse, je me suis dit (.) bon maintenant, (.) parce que moi par exemple j’adore la viande. (.) au niveau goût, j’adore. C’est vraiment une question idéologique. Je me suis dit, euhm maintenant que je suis euh: que je suis pas la seule qui qu’il y a vraiment, cette masse de la nouvelle génération qui est végétarienne ou végane, je peux me permettre de: de faire un pas: de me retirer un petit peu parce que c’est clair que au début, je disais je suis végétarienne,: c’était aussi un peu quelque chose de politique, (.) de de publicité, de expliquer le pourquoi, de sensibiliser les gens (.) et ça, j’ai je me sens plus, en devoir de le faire. Parce que j’ai vu que y a une forte euh la nouvelle génération y a une forte participation au végétarianisme et au véganisme.

Extrait 4: Léa Léa on est parti en vacances en cet été avec mon copain, (.) au Vietnam, et ils mangent énormément, (.) de viande de porc. (.) euh: y a d’la viande de porc partout hein dans les dans les enfin (.) de porc mais aussi d’autres mais surtout de porc et, (3) et c’est impossible en fait enfin des fois c’est très très dur de::: de dire euh:: enfin de trouver quelque chose déjà enfin un plat végétarien, (.) et euh: (.) et aussi on a pas envie d’faire toutes des histoires:, et euh un peu quand on- quand on mange dans une ptite euh enfin souvent c’est des familles hein c’est des restaurants familiaux, ou y a qu’un y a qu’un ou deux repas et pis: et pis on découvre notre soupe parce que enfin moi j’sais pas l’vietnamien, j’vois ma soupe, pis j’vois des trucs qui ressemblent à d’la viande j’vais pas commencer à demander,: euh si j’peux changer et tout bon après j’essaie de trier hein mais (.) après la viande de porc ben on sent la viande dans l’bouillon, (.) donc c’est vrai que (2) ça m’a pas tellement culpabilisé, (.)

### Appendix B : Transcription Conventions

**Table A1:** Transcription Conventions

Symbol	Description
,	Raising intonation
.	Fall in the intonation
?	Interrogative intonation
(.)	Small break (1 second or less)
(2) or (3)	Break (number indicates how many seconds)
:	Prolongation of the sound
Underlined word	Accentuated word
-	Interrupted word

### Appendix C: Material Used in the Qualitative Experiment

The texts used in the qualitative experiment are presented here. The order of presentation is random. They were not numbered when used in the experience. I translated them into English for this article; originals are in French. The details of the construction of these texts and their sources will be available in the doctoral dissertation that will be submitted in 2020.

#### Health, Nutrition

[Text 1] I learnt that eating too much meat and dairy products increases the risk of cardiovascular diseases, diabetes and cancer. Eating red meat and animal fats is particularly unhealthy. Before, we thought that being vegetarian was risky for health, but now even doctors recommend this diet.

[Text 2] I became vegetarian because of the ecological consequences of livestock farming. But this conversion was poorly prepared and after a few years I started to suffer from deficiencies: tiredness, hair loss, irritability… During long time, I ignored these signals, thinking that this was the right price to pay for my light conscience and a finally definitive slimness. It took me a long time to understand where this was coming from. After what, I went back to meat consumption. I am now flexitarian, to me it is the right middle between principles of reason and pleasure.

#### Environmental Impact

[Text 3] Animal farming industry largely contributes to deforestation. Livestock raised in Europe is essentially nourished with feed—mainly GM soya—produced on what once was the Amazon rainforest. Production of feed generates a diminution of available water resources and seriously damages the grounds because of the spreading of insecticides, fungicides and liquid manure. Moreover, animal farming is responsible for 18% of the greenhouse effect gas emissions. Livestock and the production of feed occupy two thirds of the entire agricultural terrain and one third of the surface of the earth, while 925 millions of people in the world suffer of hunger and could be nourished with these proteins intended for cattle.

[Text 4] If you do not eat meat anymore, in order to get enough calories you consume more other products, which also have an impact on environment. You will for instance eat more fruits and vegetables, or more soya-based products. For the same number of calories, those products need more energy, more water and entail to produce more greenhouse effect gas. Unlike what we usually think, being vegetarian is not necessarily good for the environment. You also have to consider the context; vegetarianism is not necessarily appropriate in certain regions because of the climate and the relief. In Europe, there are regions where the grounds are not appropriate for culture, and these terrains are well suited for cattle.

#### Animal Ethics

[Text 5] I eat meat only when I know where it comes from. Fortunately, there are still noble and respectful farmers who put their entire heart in the fact to produce meat, eggs, milk, respecting the animals, the environment and the consumers. Near to them, animals live a life that is preferable to that of wild animals, that are exposed to predators and endure difficult climate conditions. Of course, we kill these animals, but this death is painless and almost instantaneous, while wild animals often die after a long death throes. Additionally, milk, wool or honey are the price for the care that humans provide to cows, sheep and bees.

[Text 6] Most of the people wish animals’ well-being and refuse *a priori* to inflict them pain. But I think that, in order to be coherent, we have to renounce to all activities that implicate to make animals suffer, because none of them is obligatory. Thinking that we can conciliate animal farming and animal well-being is unrealistic. Animals are subjects-of-a-life, they possess an inherent value. This implies to stop immediately all activities in which animals are used as a mean and to completely refuse animal industry. One day will come when the idea that, in order to feed themselves, humans raised and massacred living beings and exposed their tattered flesh in shop windows, will inspire the same repugnance than cannibal meals of the savages inspired to the travelers of the XVIth or XVIIth century.

#### Pleasure, Taste

[Text 7] I adapt my diet following my principles and my desires. From time to time, I like to savor a rare prime rib of beef or roasted chicken, especially if my boyfriend prepared it. Given that I rarely eat meat, it is especially important to give myself a treat, while also choosing pieces of good quality and of ethically responsible provenance.

[Text 8] The repugnance that we spontaneously experience in front of the carcass of an animal signals that our taste for meat is not a natural tendency. As a human being, our appetite is only excited by meat, which is a transformed product, an artifice. The function of sauces, spices, perfumes is to make us forget that we consume a disgusting product. Since I thought about that, even the idea of eating meat repulses me.

#### Economic Aspect

[Text 9] Meat and fish are often the most expensive foods in a meal, in particular if you want products of good quality and which respect a few minimal environmental and ethical norms. Since I am vegetarian, I can reinvest everything I save on meat elsewhere, be it in buying foods of good quality or in other domains.

[Text 10] If I would stop eating meat, I would have to consume more other products in order to compensate, and this would weight upon my budget, especially because these products have to be of good quality in order to avoid any risk of deficiency. More people from comfortable background become vegetarian, and it is not by chance. You need time to reflect on what you will eat and plan balanced meals, take time to cook and have the means to buy substitutes to meat; not everyone can afford this.

#### Nature, Culture and Humanization

[Text 11] Exploitation of animals for their wool, their traction force and their meat allowed men to accede to civilization. Domestication and meat consumption are inseparable in human development and played an important role in the development of the brain. Meat consumption is a cultural practice that favored the mastering of fire, the production of weapons and tools and participated to the development of communication and social organization. The use of meat constitutes a symbol of the benefits of civilization.

[Text 12] It is obvious that humans are not made for meat consumption, our physiology is rather near to the one of herbivores. Our dentition is not the one of a carnivore, we do not have sharp teeth adapted to tear meat up, we have flat teeth of ruminants who chew leaves. We do not have claws to lacerate our prey. Our intestine is very long in order to allow the digestion of grass, leaves and fruits. And we do not have enough gastric juice to digest animals’ bones.
